# The need for regulation in the practice of human assisted reproduction in Mexico. An overview of the regulations in the rest of the world

**DOI:** 10.1186/s12978-021-01293-7

**Published:** 2021-11-27

**Authors:** Alma López, Miguel Betancourt, Eduardo Casas, Socorro Retana-Márquez, Lizbeth Juárez-Rojas, Fahiel Casillas

**Affiliations:** 1 Biological and Health Sciences, Metropolitan Autonomous University, 09340 Mexico City, Mexico; 2 Department of Health Sciences, Metropolitan Autonomous University-Iztapalapa Campus, 09340 Mexico City, Mexico; 3 Department of Biology of Reproduction, Metropolitan Autonomous University-Iztapalapa Campus, 09340 Mexico City, Mexico

**Keywords:** Legislation, Regulations, Reforms, Law, Human Assisted Reproduction, Mexico, Latin America, North America, Europe

## Abstract

**Background:**

The emergence of assisted reproductive technology (ART) in humans has been an important tool for the treatment of infertility. The number of treatments performed in Latin America has been increasing, and Mexico is the third country with the most assisted reproduction cycles performed in the region. However, Mexico lacks a national regulation for assisted reproduction. Therefore, it is necessary to implement regulations that allow for a safe clinical practice based on ethics which can be available to any social group.

**Main body:**

The aim of this review was to examine the existing legislation that regulates human assisted reproduction practices in Mexico, but also to examine the legal analysis of the policies, laws, and regulations in effect in some countries in Latin America, North America, and Europe. For this, seven databases were consulted, and 34 articles from 2004 to 2021 referring to the practice of ART within the legal framework and the anthropological analysis that this entails were analyzed. Eight documents were also consulted such as the Mexican General Health Law of the Official Journal of the Federation (February 7, 1984) with its last published reform (DOF 01-06-2021). And three official agency websites were also consulted. No specific legislation was found for human assisted reproduction practices in Mexico; however, assisted reproduction clinics are ruled under some agreements implemented by national organizations such as the Mexican Association of Reproductive Medicine and, at the Latin America level, the Latin America Network of Assisted Reproduction (abbreviated REDLARA in Spanish); in addition, the practice of ART is considered, although not explicitly, in the General Health Law.

**Conclusion:**

In Mexico, there is no legal regulation in charge of assisted reproduction practices, which is why there is an urgent need to establish human assisted reproduction laws without incurring discriminatory and unconstitutional acts, and at the same time, be in accordance with scientific advances. This will allow a considerable reduction in the violation of human rights.

## Plain language summary

The emergence of ART in humans has been an important tool for the treatment of infertility. It is reported that one in four couples in developing countries has fertility problems. In 2009, the International Committee for Monitoring Assisted Reproductive Technology (ICMART) established ART as "*all treatments or procedures involving *in vitro* manipulation of oocytes, sperm or embryos for the purpose of establishing a pregnancy*". The number of treatments performed in Latin America has been increasing, and Mexico is the third country with the most assisted reproduction cycles performed in the region. However, Mexico lacks a national regulation for human assisted reproduction. This has caused Mexico to become a medical tourism paradise, which increases the possibility of abuses, fraud, and clinical risks. In addition, it allows each institution offering assisted reproduction services, whether public or private, to establish arbitrary requirements for inclusion. Thus, the emergence of a regulation that allows a safe clinical practice based on ethics, which will also make this reproductive tool available to any social group, is a social need. Therefore, the aim of this review was to examine the existing legislation that regulates human assisted reproduction practices in Mexico, but also to examine the legal analysis of the policies, laws, and regulations in use in some countries in Latin America, North America, and Europe, as well as highlighting the importance of working on the establishment of regulations that allow for safe and ethically based clinical practices.

## Background

The World Health Organization (WHO) states that it is the decision of each individual and couple, according to their conscience, to determine whether they intend to have a pregnancy and if so, when they wish to have a child, as well as determining the size of the family unit. However, fertility problems may affect the possibility of pregnancy. The WHO states infertility as "*a disease of the reproductive system defined by the failure to achieve a clinical pregnancy after 12 months or more of regular unprotected intercourse*" [1]. It is reported that one in four couples in developing countries has been affected by infertility [2]. In 2012, infertility in women remained within a similar range over 20 years, from 1990 to 2010 [3]. However, in 2019 infertility increased worldwide, as it was found that the age-standardized infertility prevalence rate increased by 0.37% per year for women and by 0. 29% per year for men. Furthermore, it was observed that the highest upward trend in women occurred in countries with high sociodemographic index, and conversely, the upward trend of infertility in men occurred in countries with low sociodemographic index [4]. Infertility is not a problem limited to a region or a social group. The main factors that lead to infertility are multiple, ranging from health issues some of these conditions derived from the habits and lifestyles of a modern society as well as problems relating to the advancing age of women, derived from personal decisions, such as the delay of motherhood for professional, work, or social reasons [5]. Also, it is impossible to isolate the fact that modern society has established new forms of interaction and family conformation, expanding the concept of the idea of the formation of a nuclear family [6]. The concept of family is not limited to a heterosexual couple, but also to same-sex couples or those formed by a single father or mother with children, among others [6].

The emergence of assisted reproductive technology (ART) in humans, more than 40 years ago, has been an important tool for infertility. Some of these ARTs are in vitro fertilization (IVF), embryo transfer, gamete intrafallopian transfer, zygote intrafallopian transfer, tubal embryo transfer, gamete and embryo cryopreservation, oocyte and embryo donation, and gestational surrogacy. Assisted or artificial insemination with sperm from the woman's partner or a sperm donor is not included in ART [1, 7]. Technological and scientific advances in human assisted reproduction have enabled treatments for most infertility cases. Although the number of treatments performed in Latin America is increasing, this is the world region with the fewest treatments performed, below Europe, North America, the Middle East, and Australia/New Zealand; countries in which assisted reproduction is considered part of the public health system [8]. This limitation of ART is largely due to the lack of coverage since in Latin America countries, individuals or couples must pay for most or all treatment costs [9] favoring the use of these services to some social groups. Therefore, there is a social need for legal regulation, since Mexico, being the third country with the largest number of assisted reproduction cycles performed in Latin America [8, 10], lacks this type of regulation at a national level. This will allow safe clinical practices based on ethics and will guide the discussion on the need to make this reproductive tool available to any social group. Therefore, the aim of this review was to examine the existing legislation that regulates human assisted reproduction practices in Mexico, but also to examine the legal analysis of the policies, laws, and regulations in use in some countries of Latin America, North America, and Europe, as well as highlighting the importance of working on the establishment of regulations that allow for safe and ethically based clinical practices.

## Methods

**Fig. 1:**
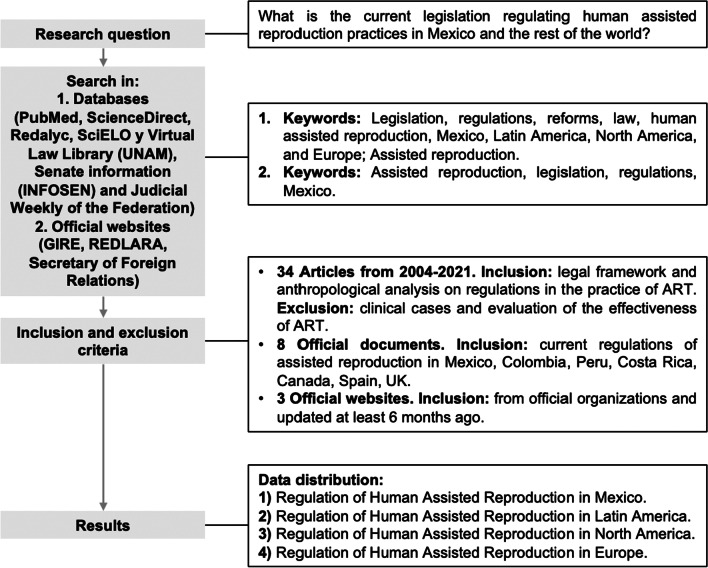
Methods diagram

The research question was, “What is the current legislation that regulates human assisted reproduction practices in Mexico and the rest of the world?” For this, seven databases were consulted: PubMed, ScienceDirect, Redalyc, SciELO, Virtual Law Library (UNAM), Senate Information (abbreviated INFOSEN in Spanish) and Judicial Weekly of the Federation, in which 34 articles from 2004 to 2021 were selected using the keywords: legislation, regulations, reforms, law, human assisted reproduction, Mexico, Latin America, North America, and Europe. Likewise, searches were made in databases of legal organizations in Mexico (INFOSEN, of the Senate of the Republic and the Judicial Weekly of the Federation), in the electronic version of the General Health Law of the Official Journal of the Federation (February 7, 1984), consulted with its last published reform (DOF 01-06-2021), as well as those in countries such as Colombia, Peru, Costa Rica, Canada, Spain, and the United Kingdom. Also, three official agency websites were consulted, with the term "Assisted Reproduction" used as a search criterion: Elected Reproduction Information Group, (abbreviated GIRE in Spanish), Latin American Network of Assisted Reproduction (known as REDLARA in Spanish), and the Secretary of Foreign Relations. The dates of consultation of all search resources were February, June, and August of 2021. Articles that pointed out the ARTs allowed and practiced within the legal framework of each of the countries were included, as well as articles that highlighted an anthropological and social analysis of the advantages and disadvantages of the regulation corresponding to the area of assisted reproduction; conversely, articles referring to clinical cases and which evaluated the efficacy of assisted reproduction techniques were excluded. From legal organization databases in Mexico, documents mentioning the current regulations of assisted reproduction were included; since those are the databases that compile the laws, regulations, and decrees in place, it was sufficient to shorten the search to “Assisted Reproduction”. Consulted websites were from official pages updated at least 6 months ago, which provided data on practices in assisted reproduction centers (ARCs) in Mexico (GIRE), as well as from a scientific and educational institution in charge of compiling information from more than 200 ARCs in Latin America. The data obtained was divided in four topics based on geographic regions: (1) Regulation of Human Assisted Reproduction in Mexico, (2) Regulation of Human Assisted Reproduction in Latin America, (3) Regulation of Human Assisted Reproduction in North America, (4) Regulation of Human Assisted Reproduction in Europe (Fig. 1).

## Results

### Structure of the Mexican government

Mexico is a Federal Republic and the Constitution currently in effect was approved by the Congress in 1917. The Supreme Power of the Federation is divided, for its exercise, into the Legislative, Executive and Judicial Powers. The Executive Power is headed by the Constitutional President of the United Mexican States, who is in charge of governing as established by law. The Legislative Power is deposited in the Congress of the Union, which is divided into the Chambers of Deputies and Senators. The Congress is in charge of issuing laws that regulate the internal structure and operation of the Mexican Republic. Finally, the Judicial Power of the Federation is formed by the Supreme Court of Justice of the Nation, which is in charge of overseeing compliance with the Constitution (the Supreme Law) and the laws [11].

### Regulation of human assisted reproduction in Mexico

Since the birth of the first girl born by IVF more than 40 years ago and the growing use of these techniques at the present, there are many countries in the world that lack regulations pertaining to this subject. Mexico is no exception, since it does not have a law that supports, protects, or regulates the operation of ARCs at a national level, consistent with the advances in science and human rights. In the absence of a legal regulation on the practice of ART, the ARCs are governed by some agreements implemented by national organizations such as the Mexican Association of Reproductive Medicine and, at the Latin American level, by the REDLARA; also, the practice of ART is also considered, although not explicitly, in the General Health Law (Table 1) [9, 10, 12].

**Table 1:** Some countries in America and their ART regulations

Art regulations			
Country	Current legislation/regulations	Year	Specifications
Mexico	None available	–	ARCs are governed under agreements established by the Mexican Association of Reproductive Medicine and the REDLARA Assisted reproduction is considered, although not specifically, within the General Health Law
Latin America			
Argentina	*Law 286.862/13*	2013	Allows national access to ART
Uruguay	*Law 19.167/2013*	2013	Inclusion of ART within the public health system Surrogacy for altruistic purposes
Colombia	*Law of 1953*	2019	Public policy for infertility prevention and treatment within the parameters of reproductive health
Peru	*Article 7, of Law 26842* *(General Health Law)*	1997	Everyone has the right to access ART treatments, but the genetic mother and the gestational carrier must be the same person
Brazil	None available	–	It is governed by an administrative agreement issued by the Federal Council of Medicine, emphasizing the ethical rules governing the use of ART
Costa Rica	None available	–	In 2000, Executive Decree 24,029-S was declared unconstitutional
Bolivia	None available	–	In 2001, draft PL 185–2001/2002 was proposed
Chile	None available	–	In 2008, Bill 6306–07 was proposed, which would penalize participants in surrogacy with imprisonment
North America			
United States	*Fertility Clinic Success Rate and Certification Act*	1992	Regulations vary by state
Canada	*The Canadian Act Respecting Assisted Human Reproduction and Related Research; AHR Act*	2004	The creation of chimeras or hybrids, germline alterations, commercialization of gametes and surrogate motherhood are prohibited

Derived from the need to regulate and control the ART performed, as well as to report the results obtained, the REDLARA emerged in Latin America in 1990. This is a scientific and educational institution responsible for compiling results and information, and establishing the standards of good practice in ARCs. This institution has more than 90% of the Latin American ARCs in its registry, being a joint endeavor of more than 15 Latin American countries, including Mexico; the second country with the most registered ARCs, only below Brazil [10, 12]. Also, Mexico is the third country with the highest number of assisted reproduction procedures performed, and babies born due to the same from 2002 to 2017 [8, 10, 12]. Therefore, the emergence of regulations for this matter has been proposed [13, 14], based on the General Health Law and Article 4 of the Political Constitution of the United Mexican States reformed in 1984, in which the right to health protection and to free reproductive decision is considered an individual guarantee [15]. This opens the possibility of making access to the necessary mechanisms to exercise the right to procreation of Mexicans, including the services of human assisted reproduction, without restriction due to sexual preferences and/or marital status [15], obligatory for the government. Article 3 of the General Health Law establishes that the sanitary control of the disposal of organs, tissues and cells is an exclusive federal matter of general health, so the possible issuance of a regulation applicable to assisted reproduction services would be based on this article [15].

### ART practices and regulations in Mexico

The ARTs performed in Mexico, such as IVF, ICSI (Intracytoplasmic Sperm Injection), gamete cryopreservation, embryo transfer, gamete donation, mitochondrial replacement therapy (by altering the genome), etc., are all allowed without any restrictions. However, the only regulated practice is gestational surrogacy. Only 4 of the 32 states of the Mexican Republic have a regulation on this matter. Tabasco (Article 92 and Chapter 6 of the Civil Code “Surrogacy and Surrogate Pregnancy”) and Sinaloa (Chapter 5 of the Family Code) recognize and regulate surrogacy in their Civil Codes. On the other hand, Coahuila (Article 491 of the Civil Code) and Queretaro (Article 400 of the Civil Code) explicitly disregard any surrogacy agreement. Likewise, the Legislative Assembly of the Federal District, now Mexico City (CDMX), presented the Surrogacy Law of the Federal District on November 30, 2010. Said regulation was never published so it is not in effect. The rest of the Mexican states have not included surrogacy within their legislation in the corresponding matters (Table 2) [16].

**Table 2:** ART practices and regulations in Mexico

ART practices and regulations in Mexico		
ART	Current legislation/regulations	Specifications
In Vitro Fertilization (IVF) (Including Intracytoplasmic Sperm Injection, ICSI)	None available	None available
Embryo transfer	None available	None available
Gamete intrafallopian transfer	None available	None available
Zygote intrafallopian transfer	None available	None available
Tubal embryo transfer	None available	None available
Gamete and embryo cryopreservation	None available	None available
Oocyte and embryo donation	None available	None available
Mitochondrial replacement technique (MRT)	None available	None available
Gestational surrogacy	Civil and Family codes of the states of Tabasco, Sinaloa, Coahuila, and Queretaro	Tabasco and Sinaloa recognize and regulate surrogacy Coahuila and Queretaro explicitly disregard any surrogacy agreement

### Assisted reproduction practices in Mexico

Despite the absence of legal regulation, the Mexican government has provided human assisted reproduction services in public institutions of its National Health System, such as the Mexican Institute of Social Security (abbreviated IMSS in Spanish), the Institute of Security and Social Services for State Workers (known as ISSSTE in Spanish) and the Isidro Espinosa de Los Reyes National Institute of Perinatology. At the same time, private clinics of human assisted reproduction offer a wide variety of treatments which are not subject to the same restrictions as they are in the public sector although they employ management and marketing schemes at higher costs [13, 16].

The wide variety of treatments offered has allowed even foreign professionals to perform therapies that are not allowed in other countries, as was the case of a Jordanian couple who resorted to the mitochondrial replacement technique (MRT; specifically, maternal spindle transfer) and embryo transfer in Mexico, which resulted in the birth of a healthy child [17]. In this regard, one of the scientists involved in the procedure indicated that the manipulation-derived embryo transfer treatment was performed in Mexico due to a lack of rules. Palacios-González and Medina-Arellano [18] claim that, under their interpretation of the law and with the information available on the case, the research team that performed this practice violated Article 56 of the Regulations of the General Health Law, which establishes that research on assisted fertilization is only permitted when it is intended to solve infertility problems. Since this practice is not explicitly permitted or prohibited legally, it may fall into an interpretation gap, where the practice performed was not legal, since the woman treated in the study was not infertile (two naturally conceived children died at the age of 6 years and 8 months, respectively, due to Leigh syndrome) [18].

### Requirements for the access to public assisted reproduction practices in Mexico

Of the 42 centers registered in the REDLARA, more than half of them belong to the private sector [12]. However, access to reproduction treatments performed in these institutions continues to have major limitations, as the lack of regulations in the country leaves open the possibility of incurring in abuses and human rights violations, and there is a lack of legal protection for the medical workers involved. This also lends itself to each institution, whether public or private, determining its criteria for inclusion, which in many cases can be discriminatory and arbitrary. Such is the case of allowing access to ART only to married couples (Women's Specialties Clinic, ISSSFAM and ISSSTE’s 20 de Noviembre National Health Center, although the latter also allows for cases of cohabitation) and in some cases only to a man and a woman (Isidro Espinosa de los Reyes National Institute of Perinatology) or with a maximum of previous children (Women's Specialties Clinic, ISSSFAM and ISSSTE’s 20 de Noviembre National Health Center) [16]. Another condition is the age limit. In women, the age range varies from 40 years old or younger (Women's Specialties Clinic, ISSSFAM), 36–35 years old or younger (ISSSTE’s 20 de Noviembre National Health Center and Isidro Espinosa de los Reyes National Institute of Perinatology, respectively) and between 19 and 37 years old (Mónica Pretellini Maternal Perinatal Hospital, in the State of Mexico). In men, the age range varies from 50–55 years or younger (Women's Specialties Clinic, ISSSTE’s 20 de Noviembre National Health Center and Isidro Espinosa de los Reyes National Institute of Perinatology, respectively) [16]. In addition, it is important to guarantee the health of the beneficiaries of public programs. Couples must be emotionally stable persons without life-threatening diseases during the pregnancy, or who suffer from diseases that may be transmissible, as well as the presence of infertility or the loss of two or more consecutive pregnancies, and in heterosexual marriages, having had unprotected sex for at least 1 year previously without having achieved pregnancy (Table 3) [16].

**Table 3:** Requirements for the access to public Assisted Reproduction Practices in Mexico (modified from GIRE)

Requirements for the access to public assisted reproduction practices in Mexico				
Requirements	Women's Specialty Clinic (ISSSFAM)	20 de Noviembre National Health Center (ISSSTE)	Isidro Espinosa de Los Reyes National Institute of Perinatology	Monica Pretellini Maternal Perinatal Hospital (State of Mexico)
Women's age	< 40 years	< 36 years	< 35 years	Between 19 and 37 years
Men's age	< 50 years	< 55 years	< 55 years	Between 19 y 55 years
Health	Emotionally stable persons, without diseases that can be transmitted, or which can be life-threatening during the pregnancy	People without transmissible diseases	With infertility	With infertility or the loss of two or more consecutive pregnancies
Marital status	Legally constituted couples	Married or cohabiting couples	Heterosexual couples under any type of cohabitation	"A female and her male partner", without specifying marital status
Children	Couples with less than two living children with their current partner	Maximum one previous child	No requirements	No requirements

### The incurrence of discriminatory acts

Several cases in Mexico have set a precedent for discriminatory acts committed by some institutions. Some of these cases are related to discriminatory acts based on the woman’s age. Due to the legal system established in Mexico, a judge determines the facts for each case and, with them, the application of provisions related to it, giving a final resolution [19, 20]. In 2017, the GIRE reported several cases registered, documented, and litigated from 2015 to 2017. These included the documentation of cases of pregnant women who were defrauded by intended parents due to a lack of legal contracts drawn by some surrogacy agencies, as well as by intended parents, and litigation of international parents who were denied registration of their children due to the failure of the Civil Registry system to adapt to the new types of affiliations demanded by modern society [16]. Some judges have ruled the age limit imposed by some institutions as a discriminatory act, arguing that the success of ART is not associated exclusively with the age of the patients, but also by their reproductive capacity. Other judges have decided not to pronounce discrimination in the requirements for admission to assisted reproduction practices, thus requesting the intervention of the Supreme Court of Justice of the Nation (abbreviated SCJN in Spanish) [16]. This shows that, in the absence of regulations, resolutions are based on the interpretations of judges and public officials. Even though these processes are quite slow and open to interpretation, institutions such as the Judicial Power of the Nation, the National Council to Prevent Discrimination (known as CONAPRED in Spanish) and the National Human Rights Commission (abbreviated CNDH in Spanish), can help regulate the requirements for the access to the ARTs that may incur in discriminatory acts.

### Current reforms in the field of human assisted reproduction

According to some experts, the 2016 approval of a reform to the Civil Code of the State of Tabasco rushes in some respects such as invasion of competencies, discrimination, and legal insecurity. For example, pregnant women must be between 25 to 35 years old, and the gestation contract must be signed by the contracting mother and father with the pregnant woman (Article 380 BIS 2), the contract will be annulled for intervening agencies, offices and third parties (Article 380 BIS 4). Access is only for Mexican citizens, the implantation has a limit of up to two embryos, and any contract must be approved by a competent judge (Article 380 BIS 5) [16, 20–22]. In 2013, agreements in the matter of human assisted reproduction were introduced in the Civil Code of the State of Sinaloa. These considered restrictions which had not previously been taken into account in the State of Tabasco and resulted in achieving that the State of Sinaloa did not become a destination for reproductive tourism as is the case of Tabasco [16, 20, 22].

In 2016, the Chamber of Deputies issued a regulation on human assisted reproduction which was based on an initiative previously presented initiative. It considers the requirement of a medical diagnosis of infertility to permit access to ART, prohibits the use of sperm donation (not applicable for egg donation), the restriction for gamete donation and the approval of spouses of married women who wish to undergo any assisted reproduction procedure [16]. In 2018, a new initiative to the General Health Law regarding human assisted reproduction was presented before the same relevant commissions, and it establishes reproduction achieved through ovulation induction, controlled ovarian stimulation, ovulation triggering, and techniques such as intrauterine, intracervical, or intravaginal insemination with semen from the husband, partner, or donor. In addition to all treatments or procedures that include manipulation, both of oocytes and sperm or embryos, for the establishment of a pregnancy (Art. 71 bis, I and II). However, up to date, it has not been approved by the incumbent authorities in force [23].

### Regulation of human assisted reproduction in Latin America

Most Latin American countries do not have regulations that specifically control ART. Some rules with relevance in the matter consist of general principles based on Civil and Criminal Codes or which are mentioned in their Constitution [24]. Although there are differences between countries, economic inequality and the high influence of Catholicism have a significant impact on ART regulations in Latin American countries [7]. In 2014, countries such as Chile, Colombia, Ecuador, Peru, Uruguay, Venezuela, Argentina, and Brazil, reported the most ARTs used, and the latter two countries reported a higher number of cycles performed, with fertilizations being the most recurrent techniques with IVF/ICSI [12].

### Latin America countries with ART regulations

Only Argentina (Law 286.862/13, issued by the Chamber of Deputies) and Uruguay (Law 19.167/2013, issued by the Senate), have specific regulations on the subject which were issued in 2013. These laws accredit ARTs and stipulate the requirements to be met by public and private institutions for the practice of such procedures [24]. Argentina regulated ARTs for the first time in 2010. It recognizes the right of a person to procreation, and categorizes infertility as a disease. In addition, the Law that arose in 2013, broadens access to ARTs to any adult person, regardless of age, marital status and whether they present pathological infertility, thus allowing national access to ARTs [19, 24]. In 2013, Uruguay approved Law 19.167/2003, which addresses the inclusion of ARTs within the Uruguayan public health system (Art. 3). Likewise, it mentions that surrogacy should only be for altruistic purposes [19, 24].

In Colombia, according to Article 42-6 of the Colombian Constitution, children born naturally or through ART have the same rights and obligations. In 2009, a legal precedent (T-968/2009) emerged concerning surrogate motherhood, to protect the rights of newborns and surrogate women. In 2014, it was ruled that same-sex couples can adopt a child when one of them is the biological parent of said child [19]. Finally, in 2019, Law 1953 was established, which is the agreement "whereby the guidelines for the development of public policy for the prevention of fertility and its treatment within the parameters of reproductive health" [25]. In Peru, Article 7 states that everyone has the right to access ART treatments, but the genetic and gestational mother must be the same person. There is no specific legislation on surrogacy, and the Health Law (Law 26,842) partially addresses the issue (Table 1) [26].

### Latin America countries without ART regulations

Brazil does not have a specific law for ARTs; however, it is regulated under an administrative agreement issued by the Federal Council of Medicine, which highlights the ethical norms governing the use of ARTs are accentuated. Costa Rica is the only country in the world in which IVF was concretely prohibited, through an appeal of unconstitutionality appeal against Executive Decree (24029-S), resolved in 2000, in which the right to life was challenged, due to the argumentation which considers embryos as human beings [18, 27]. Bolivia is one of the countries that does not have specific legislation on surrogacy. In 2001, Bill PL 185-2001/2002 was proposed in the Bolivian National Congress. The law was not clear on whether the commercialization of surrogacy was allowed or not; however, it was intended to address the issue of infertility and it raised the written consent of all parties involved before initiating any fertilization procedure [19]. In 2008, Chile presented Bill 6306-07 which contained a single article (Article 23), stating it would penalize the participants in surrogacy with jail; this bill has not been approved yet. Because of this, judges must intervene according to a test that defines the person who has given birth to the baby as the biological mother (Article 183 of the Chilean Civil Code) (Table 1) [19, 24]. Surrogacy is the practice that is most regulated in Latin American countries, unlike other ARTs. In countries such as Chile and Colombia, it has been attributed the slow progress of a normative regulation has been attributed to the strong social influence of the Catholic Church, which extends to public policies and national legislation [7, 19, 24].

### Regulation of human assisted reproduction in North America

#### ART regulations in the United States of America

In 1992, the Fertility Clinic Success Rate and Certification Act was created in USA; its aim was to standardize the reporting of ART success rates throughout the country. This was to be done through the joint work of various organizations such as the Society for Assisted Reproductive Technology (SART), American Society of Reproductive Medicine (ASRM), Centers for Disease Control (CDC), and the National Institutes of Health (NIH), among others, which are responsible for reporting data on assisted reproductive treatment cycles from ART clinics in the USA each year [28–31].

Among the practices they regulate are those already established such as IVF and experimental techniques that have been able to transcend to clinically accepted treatments due to the promising results obtained, such as cryopreservation of oocytes, embryos, and ovarian tissue [24, 29, 30]. In addition to the collection of clinical outcomes, the regulation of ART practices achieved by the Fertility Clinic Success Rate and Certification Act, some aspects were also considered, such as the barriers that limited access to ART procedures in certain social groups, in addition to the ethical and legal implications regarding processes such as preimplantation genetic test (PGT), as well as gamete and embryo donation [24, 30, 31]. Regarding the latter, the National Organ Transplant Act of 1984 allows the commercialization of sperm and eggs for specific purposes, so financial compensation to egg donors is accepted [29, 30]. Also, it considers issues such as the use of egg donation from family members, oocyte donation to women of advanced reproductive age, recovery and posthumous use of oocytes, information to offspring about their conception, the establishment of paternity/maternity (considering surrogacy), as well as disclosure of medical errors made, informed consent even for donation for research purposes and on the rights and obligations in gamete donation [30]. It is important to note that these regulations vary at the state level. Seven states prohibit human cloning for reproductive and research purposes, eight more prohibit only reproductive cloning. Some states prohibit commercial surrogacy or regulate surrogacy arrangements, as well as sperm, egg, and embryo donation (Table 1) [30, 31].

### ART regulations in Canada

In Canada, the Canadian Act Respecting Assisted Human Reproduction and Related Research (AHR Act) came into effect in 2004. This law is based on ethical and social considerations, which prohibits the practice of a variety of technologies including the creation of chimeras or hybrids, alterations of human germlines, as well as the commercialization of gametes (eggs and sperm) and surrogate motherhood, in which, contrary to the regulation in the USA, participation for profit in these acts would be considered criminal offenses (Table 1) [31–33]. The development of this law took into account bioethical interests, medicine, women's health, feminist activism (which achieved an anti-commercialization stance, notable in the AHR law), the rights of people with disabilities, services for immigrant women, theology, political advocacy, law, and human rights. The support and objection of health professionals to the proposals for these regulations were also considered. In addition, it is mentioned that a strong influence of the policies of nations close to Canada, such as the United Kingdom and the USA, played an important role in the structuring of the AHR law [32].

In March 2013, with the passage of federal budget Bill C-38, Canada's Human Assisted Reproduction Program was closed. That program was responsible for administering and enforcing the AHR Act. Among the most significant changes made by Bill C-38 was the inclusion of the importation, distribution and clinical use of donated oocytes and sperm into the criminal framework of the AHR Act. This moved the right to regulate donors from the Food and Drugs Act to the AHR Act, resulting in the attribution of criminal liability to health professionals, who, if not subject to the regulations, could be sentenced to up to 5 years in prison and/or a fine of $250,000 [34]. However, this also meant an advance in the inclusion of social groups that had been segregated under the old regulations, such as lesbian, gay, bisexual, trans and queer Canadians. For example, gay men were excluded from donating their sperm unless they were in a sexual relationship with the recipient or if they received special permission from the Minister of Health. The use of donor sperm for procreation was more difficult for lesbians and single women, as they were subject to strict regulatory requirements, while women who used the sperm of their sexual partners had an easier process [34, 35]. Before Bill C-38, there were no regulations for the regulation of importation and distribution of oocytes [36], which set the tone for the implementation of rigorous testing for their handling.

In 2018, a workshop entitled Consensus Statement: gene editing, genetic testing, and reproductive medicine in Canada was held in Ottawa, Canada (Consensus Statement: Gene Editing, Genetic Testing, and Reproductive Medicine in Canada). It aimed to propose a restructuring of the AHR Act (amended in 2013) [35, 37], in order to take into account the interests of physicians and researchers for the promotion of medical and scientific innovation through the adaptation of in vitro and in vivo research that is prohibited, such as gene editing research for the correction of genetic mutations, somatic cell nuclear transfer (SCNT), the use of embryos produced in vitro that will be discarded and that could later be used for research to expand the knowledge of processes such as early embryonic development and developmental disorders, as well as research on mitochondrial replacement therapy (through genome alteration), research on the development of human organs, the origin of human diseases and the study of human primordial germ cells (through the creation of chimeras) [35, 37].

### Regulation of Human Assisted Reproduction in Europe

#### Common ART regulations in the European Union

In the European Union (EU), 43 countries have a legal framework regarding human assisted reproduction. Almost all these countries, except for Albania, Bosnia and Herzegovina, Ireland, Romania, and Ukraine [38], have specific legislation and public health legislation in this area. Even though most of these legislations converge in similar characteristics between countries, each country governs and stipulates its specific conditions for the use of ARTs. At present, there have been important reforms in the legislation, which considers the needs of today's society have been considered, including social groups that a few years ago were still excluded from the use of ART, such as single women, lesbians, and same-sex couples. However, some countries, such as the Czech Republic, France, Italy, Poland, Slovakia, Slovenia, Switzerland, and Turkey, continue to limit access to ART exclusively to heterosexual couples with a verifiable pathological diagnosis of infertility, thus excluding single women and lesbians [38–43]. In most countries, the minimum age for sperm donors is 18, with a maximum of 40. Likewise, 30 countries have established the condition of conceiving a maximum of five infants from the same donor. In the case of women egg donors, a minimum age of 18 and a maximum of 35 have been established in most countries for egg donors [38]. Sex selection of the embryo by PGT-A testing is not allowed in any country, except for the detection of sex chromosome-related diseases, in which case, some countries allow the test to be performed [43]. The freezing of gametes, particularly oocytes, is permitted in all countries under medical considerations; for example, for the preservation of fertility before beginning chemotherapeutic treatment [38, 39].

In countries like Denmark, Sweden, Netherlands, France, Belgium, Czech Republic, and Slovenia, patients are provided public financial assistance by their respective governments [38]. Funding is conditioned on the maximum age of the woman, on whether she has had children previously, and on having received public support for previous treatments. Only four EU countries, including Ireland, do not have such financial assistance. This brings ease of access to AMR treatment for most people in these countries, regardless of socioeconomic status. However, this has also led to longer waiting times (between 12 and 24 months) in public centers, as compared to private centers, resulting in cross-border reproductive tourism. For example, French and Italian citizens travel to other countries such as Greece, Spain, and Belgium. Sperm and oocyte donation are the practices most in demand by these citizens, most of whom are same-sex couples, single women, or heterosexual couples who did not qualify for the procedures in their country, since access to AMR in France and Italy was allowed only for the resolution of sterility or infertility problems in adult heterosexual couples of potentially fertile age, which also proved to be married or living together, in addition to both partners being alive [38, 40, 42–44].

### Specific ART regulations in the European Union

In Spain, the first law for ART was approved in 2006 (Law 14/2006) [44]. In 2007, the approval of Biomedical Law 14/2007, led to the creation of the National Commission for Assistance to Human Reproduction, a committee that regulates ART in that country (Table 4) [30, 38]. In Spain, the "menopausal age" is considered as a limit, and surrogacy is not recognized [38]. Some specifications of the laws referred to in each country are described in Table 4.

**Table 4:** Some countries in Europe and their ART regulations

Art regulations			
Country	Current legislation/regulations	Year	Specifications
Spain	*Human Assisted Reproduction Technique Law 14/2006* *Biomedical Law 14/2007*	2006 2007	It prohibits reproductive cloning, transfer of more than three embryos per reproductive cycle, germline modification, non-medical sex selection and the use of PGT for non-medical purposes Surrogacy is not recognized
United Kingdom	*Surrogacy Arrangement Act* *The Human Embryology and Fertilization Act* *Human Reproductive Cloning Act* *Human Fertilization and Embryology (Mitochondrial Donation) Regulations 2015, Nº 572*	1985 1990 1990 2005	Prohibit reproductive cloning, germline modification, non-medical sex selection, commercial egg and sperm donation, and commercial surrogacy. Regulates the use of donor gametes, assisted fertilization, PGT, gamete and reproductive tissue banking, and human embryo research
Italy	*Law No. 40. Medically Assisted Procreation Law*	2004	2009: the Constitutional Court declared as unconstitutional the maximum limit of embryos to be produced and transferred for each cycle (three, according to the original version) 2014: the Constitutional Court allowed heterologous assisted reproduction 2015: the Constitutional Court granted the right to access ART to couples who are fertile but carriers of genetic diseases
France	*Law on the Donation and Use of Elements and Products of the Human Body, Medically Assisted Procreation, and Prenatal Diagnosis, No. 94–654* *Bioethics Law No. 2004–800*	1994 2004	The Bioethics Law prohibits reproductive and research cloning, germline modification, non-medical sex selection and surrogacy PGT is only allowed when a parent or close relative has a serious genetic disease
Germany	*Federal Embryo Protection Law* *Adoption Brokerage Law* *Guideline of the German Federal Medical Chamber*	1990 2006 2006	Reproductive and research cloning, gamete donation, creation of hybrid embryos, cryopreservation of fertilized eggs, sex selection (except sperm selection for the prevention of certain sex-related genetic disorders), PGT and all forms of surrogacy are prohibited
Switzerland	*Federal Law on Medically Assisted Reproduction* *Federal Act on Research Involving Embryonic Stem Cells* *Federal Law on Medically Assisted Reproduction*	1998 2003 2004	Reproductive and research cloning, egg and embryo donation, creation of an embryo for research purposes, creation of a hybrid embryo, germline modification, PGT, non-medical sex selection and surrogacy are prohibited. The destruction of cryopreserved gametes and embryos is mandated after 5 years

The laws governing assisted reproductive practices in the UK date back to the 1985 *Surrogacy Arrangements Act*, and to the *Human Embryology and Fertilization Act* and the *Human Reproductive Cloning Act of 1990*. Nowadays, with its suggestion in 2005 and its last regulation in 2015, the *Human Fertilization and Embryology (Mitochondrial Donation) Regulations 2015, No. 572*, has established the Human Fertilization and Embryology Authority (HFEA) as being responsible for licensing ARCs (Table 4) [45]*.* HFEA limits the transfer of 1–2 embryos per reproductive cycle in women under 40 years of age and a maximum of 3 embryos in women over 40 years of age [30, 45]. Although anonymity is also applicable for donation recipients, when the children born through donation exceed a defined age, they can have access to the donors' identity [38]. The legal practice of MRT has only been explicitly allowed in the UK since 2015 after both houses of parliament accepted proposals made by the Department of Health [18].

In Italy, the *Medically Assisted Procreation Law No. 40* was approved in 2004. However, there have been restructurings to its system, as it was in 2009 when the Constitutional Court declared as unconstitutional a maximum limit of embryos to be produced and transferred for each cycle (three, according to the original version) [30]. In 2014, the prohibition on donor insemination and gamete donation was declared unconstitutional, making heterologous assisted reproduction legitimate again, although with the limitation of being applied exclusively in different-sex couples, married or cohabiting, and only in women of childbearing age [42, 43]. In 2015, the right of access to AMR was granted to fertile couples who were carriers of genetic diseases, thus allowing the use of PGT-A in these couples (Table 4) [46].

In France, the *Law on the Donation and Use of Elements and Products of the Human Body, Medically Assisted Procreation and Prenatal Diagnosis, No. 94–654* was approved in 1994 and, in 2004, the *Bioethics Law No. 2004-800* was also approved (Table 4). An important reform to the legislation considers the elimination of anonymity of sperm and oocyte donors. Since the laws of 1994, the law of 2004, and the current law enacted in 2011, is considered the voluntary nature of the donation and the anonymity of the donor, in addition to respect for human dignity and the non-commercialization of such practice are taken into account, but with the possibility of retribution for expenses that may be generated by the donor, as well as any activity that generates a profit is legally banned for all involved [32, 38, 39, 41, 42]. Also, egg cryopreservation for non-medical purposes is not allowed [30, 32]. The *Federal Embryo Protection Law (1990),* the *Adoption Brokerage Law (2006)* and the *Guideline of the German Federal Medical Chamber (2006)* are the regulations governing ART practices in Germany (Table 4). There are no legal limits for egg donation. It is legal to donate sperm for IVF and intrauterine insemination; however, egg donation is prohibited [30, 32]. Also, egg cryopreservation for non-medical purposes is allowed [30]. The *Federal Law on Medically Assisted Reproduction (1998),* the *Federal Act on Research Involving Embryonic Stem Cells (2003)* and *Federal Law on Medically Assisted Reproduction (2004)* are the treaties under which assisted reproductive practices are governed in Switzerland (Table 4) [30, 32]. In most EU countries it is legal to donate sperm for IVF and intrauterine insemination; however, egg donation is prohibited in Switzerland [30]. Also, egg cryopreservation for non-medical purposes is allowed [30, 32].

## Discussion

No specific legislation was found for human assisted reproduction practices in Mexico, but it was found that ARCs in Mexico are governed by some agreements implemented by national organizations (Mexican Association of Reproductive Medicine), and at the Latin America level by the REDLARA. In addition, it was found that reproductive health is considered within the General Health Law and in Article 4 of the Political Constitution of the United Mexican States, which mentions the free reproductive decision-making of all Mexicans. However, Mexico does not have a law that supports, protects, and regulates the practices in ARCs at the national level. The ART practices carried out are standardized through the parameters created by organizations such as REDLARA, which establish good practice standards in ARCs in Mexico and Latin America. ART practices performed in Mexico include IVF, ICSI, gamete freezing, embryo transfer, and gamete donation, among others. Some practices performed in Mexico are not allowed in most countries of the world, such as MRT and surrogacy, the latter being the only practice explicitly included in the Civil Code of four Mexican States. The application of regulations for the use of ART in Mexico, based on the ethical principles of science and social responsibility, could ensure secure access to ARTs for the entire socioeconomic and cultural spectrum, making it possible to protect the public’s health without limiting the scientific progress that these practices bring with them. It has been considered that social, cultural, and religious factors established in different countries, including Mexico, limit the possible regulations and their application, especially those concerning treatments related to gamete donation and surrogacy. The latter practice being the one that is regulated to a greater extent in most countries, due to the risk of human rights violations.

It is necessary to consider the problems related to human assisted reproduction from a transnational perspective because they arise as a result of technological and cultural progress, and from need for the laws that regulate them to adapt to these advances. It is also essential to consider the new family structures in order not to incur in discriminatory and unconstitutional acts that restrict access to ARTs only to a certain group of people, since they are true family structures that participate, collaborate, and interact in all personal, social, cultural, and political spaces [21, 47]. In addition to being an essential human right, the formation of a family and access to the benefits that scientific advances bring, regardless of marital status, sexual orientation, gender, or age should be preserved [47].

It is important to emphasize that a lack of regulations can cause countries to become an assisted reproduction destinations, and even medical tourism paradises, as in the case of Mexico, which also increases the possibility of abuses, frauds, and clinical risks, since procedures are cheaper than in other countries. Also, it allows each institution offering assisted reproduction services, whether public or private, to establish its requirements for inclusion, which can be arbitrary, in addition to establishing its costs for each of the ARTs offered.

Specialists in the field have put forward some recommendations of elements for a model legislation on human assisted reproduction. Some of these recommendations are to avoid the criminalization of the parties involved in the agreements, as well as to avoid discrimination based on arbitrary criteria such as nationality, age, sexual orientation, and marital status in the access to practices [20, 21, 47, 48], in addition to assuring quality and confidential health services, as well as having independent legal representation that guarantees the protection of the persons requesting the services and of the health professionals involved [15, 21]. The structuring of legislation with a gender perspective that protects the interests of the women involved, particularly in cases of surrogacy, is also recommended [48].

Although this article points out the need that exists for the creation of specific and explicit legal regulations in the area of assisted reproduction in Mexico, there are still limitations to a deeper investigation into the subject, since it is still not clear which treatments are allowed in practice due to the lack of a source that compiles this information promptly, as each ARCs, both public and private, manages its catalog of ARTs offered and its criteria for inclusion and exclusion for access to these ARTs.

## Conclusion

In Mexico there is an urgent need to regulate and establish human assisted reproduction laws without incurring in discriminatory and unconstitutional acts and, at the same time, being in accordance with scientific advances. This will allow a considerable reduction in the violation of human rights. Because of this, there is a need to establish regulations that help to homogenize the procedures allowed in public and private ARCs, as well as the criteria for inclusion and exclusion of the population that can make use of these ARTs, all within a legal framework that does not violate human rights and does not incur in acts of arbitrariness, thus seeking the common good of both patients and health professionals, and allowing scientific progress in the same way. For this reason, it is recommended that more multidisciplinary studies be carried out in which not only legal specialists are involved, but also health professionals and social specialists who have the necessary perspective to guide the conversation towards the emergence of these areas of opportunity.

## Data Availability

The databases used during the current study are available from the corresponding author on reasonable request.

## Abbreviations

IVF: In vitro Fertilization
ICSI: Intracytoplasmic Sperm Injection
ART: Assisted Reproductive Technology
ARCs: Assisted Reproduction Centers
SCNT: Somatic Cell Nuclear Transfer
PGT-A: Preimplantation Genetic Testing for Aneuploidy
REDLARA*: Latin American Network of Assisted Reproduction
IMSS*: Mexican Institute of Social Security
ISSSTE*: Institute of Security and Social Services for State Workers
MRT: Mitochondrial Replacement Technique
CONAPRED*: Judicial Power of the Nation, the National Council to Prevent Discrimination
CNDH: National Human Rights Commission
CDMX: Mexico City
EU: European Union
SART: Society for Assisted Reproductive Technology
ASRM: American Society of Reproductive Medicine
CDC: Centers for Disease Control
NIH: National Institutes of Health
AHR Act: Canadian Act Respecting Assisted Human Reproduction and Related Research
Bill C-38: Federal Budget Bill C-38
*: Abbreviated in Spanish
